# Supplementary material for: Ultra-brief intervention for problem drinkers: research protocol
Source: BMC Public Health. 2008 Aug 26;8:298. doi: 10.1186/1471-2458-8-298 (PMC2528012; doi:10.1186/1471-2458-8-298)

Your choices about drinking

Are you concerned about your drinking? Take a look at the options below to see if there is anything you would like to do right now. It is your choice.

- ○ My drinking is fine for now. I will continue to watch how much I drink.
- ○ I will think about changing my drinking. Did you know that 75 per cent of people change their drinking on their own?
- ○ I will reduce my drinking to a low-risk level. This means I will drink no more than two drinks a day, with a weekly maximum of 14 drinks for men and nine drinks for women. Most people watch their drinking – they put limits on how much, when and where they drink. To avoid intoxication, they drink slowly, waiting at least one hour between drinks. They have food and non-alcoholic drinks along with alcohol. They have at least one or two days a week without any alcohol. And they don’t drive or operate heavy equipment after drinking. Women who are pregnant, trying to conceive, or are breast-feeding are encouraged not to consume any alcohol.
- ○ I will contact the Drug and Alcohol Registry of Treatment (DART) to find out about other programs and groups that can help me or someone that I am concerned about. (Call toll free 1 800 565-8603.)

For more information on addiction and mental health issues, or a copy of this brochure, please contact CAMH’s R. Samuel McLaughlin Addiction and Mental Health Information Centre:

Ontario toll-free: 1 800 463-6273
Toronto: 416 595-6111

To order multiple copies of this brochure, or other CAMH publications, please contact:
Publication Services
Tel.: 1 800 661-1111 or 416 595-6059 in Toronto
E-mail: publications@camh.net

To make a donation, please contact:
Centre for Addiction and Mental Health Foundation
Tel.: 416 979-6909
E-mail: foundation@camh.net

If you have questions, compliments or concerns about services at CAMH, please call our Client Relations Co-ordinator at:
Tel.: 416 535-8501 ext. 2028

Visit our Web site at: www.camh.net

Copyright © 2000 Centre for Addiction and Mental Health

Disponible en français :
416 595-6111

camh
Centre for Addiction and Mental Health
Centre de toxicomanie et de santé mentale

A Pan American Health Organization /
World Health Organization Collaborating Centre
Affiliated with the University of Toronto

2682/07-06 P399

Evaluate your drinking

Would you like to know how your drinking compares to other Canadians?

Take this simple test to find out.

camh
Centre for Addiction and Mental Health
Centre de toxicomanie et de santé mentale

1 What was your drinking like during a typical week in the past year?

List roughly how many drinks you have on each day of a typical week and add up the total:

Monday	
Tuesday	
Wednesday	
Thursday	
Friday	
Saturday	
Sunday	
**TOTAL**	

Be sure to estimate the number of "standard" drinks you usually have (it is important when you compare your drinking to other Canadians). Each of the drinks on the chart below have the same amount of alcohol in them and will all affect you in the same way.

One standard drink is:

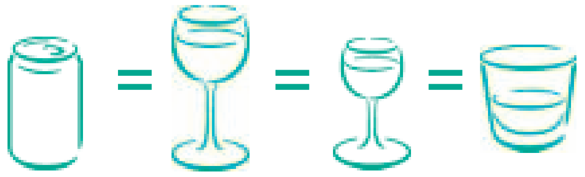

12 oz. Beer (5% alcohol) = 5 oz. Wine (10–12% alcohol) = 3 oz. Fortified Wine (16–18% alcohol) = 1.5 oz. Liquor (40% alcohol) (1.5 oz. overproof liquor is about two standard drinks)

2 Now compare your weekly total to that of other Canadians

How does your weekly average compare? Look at the pie charts below to find where your drinking fits with the rest of the adult population. For example, if you are a male who drinks 15 **standard** drinks per week, you drink more alcohol than 90 per cent of other men in Canada do.

Drinks per week

Men

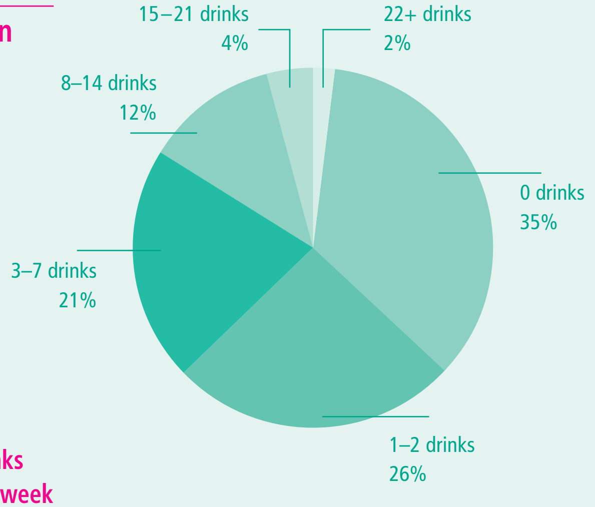

Drinks per week

Women

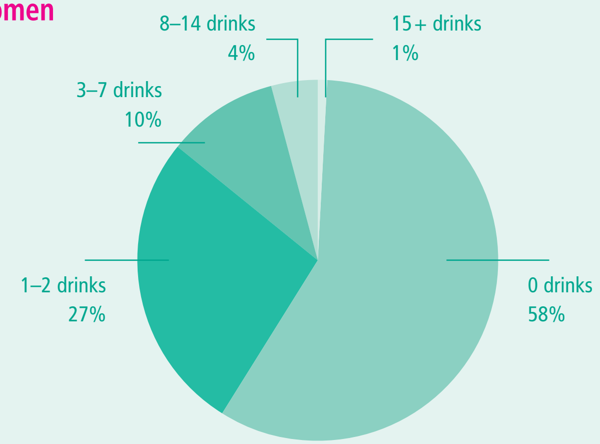

3 Risky drinking

A recent national survey looked at how much people drank in a week and how their drinking was affecting different areas of their lives. People were asked about their physical health, outlook on life, friends/social life, relationships with their spouses or partners and children, home life, financial position and work or studies. Not surprisingly, the results showed that the more people drank in a week, the greater the chance that the drinking was affecting more and more areas of their lives.

How likely are you to have problems as a result of your drinking? Look at the chart below to see where you fit.

Chance of negative consequences related to number of drinks per week

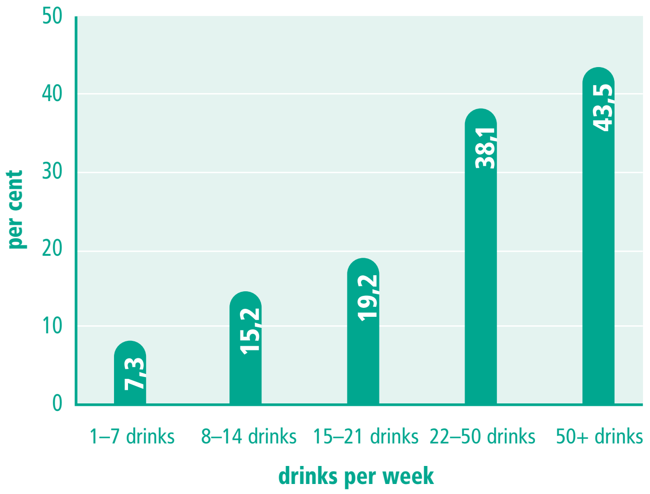

Supplement: Additional file 1 — An electronic copy of the intervention pamphlet, Evaluate Your Drinking, to be used in the research trial. [file 1471-2458-8-298-S1.pdf]
