# Supplementary material for: Ultra-brief intervention for problem drinkers: research protocol
Source: BMC Public Health. 2008 Aug 26;8:298. doi: 10.1186/1471-2458-8-298 (PMC2528012; doi:10.1186/1471-2458-8-298)
Supplement: Additional file 2 — An electronic copy of the control condition pamphlet, Do you know .... Alcohol, to be used in the research trial. [file 1471-2458-8-298-S2.pdf]

Physical dependence involves tolerance to alcohol's effects, and withdrawal symptoms when drinking is stopped. As people develop tolerance, they need more and more alcohol to produce the desired effect. People who are physically dependent on alcohol can develop withdrawal symptoms, such as sleeplessness, tremors, nausea and seizures, within a few hours after their last drink. These symptoms can last from two to seven days and range from mild to severe, depending of the amount of alcohol consumed and the period of time over which it was used. Some people experience *delirium tremens,* or "the DTs," five to six days after drinking stops. This dangerous syndrome consists of frightening hallucinations, extreme confusion, fever and racing heart. If left untreated, severe alcohol withdrawal can result in death.

Treatment for alcohol dependence usually begins by treating withdrawal symptoms, but most people will need additional treatments to help them stop drinking. Even after long periods of abstinence, a person may continue to crave alcohol, and may begin to drink again. Treatment may include residential or outpatient treatment, individual or group therapy, self-help or mutual help groups, such as Alcoholics Anonymous, and certain medications, such as naltrexone. Some people respond well to one form of treatment, while others do not. There is no single most effective treatment approach.

What are the long-term effects of drinking alcohol?

How alcohol affects you in the long term depends on how much and how often you drink.

For middle-aged and older adults, as little as one drink of alcohol every other day can help protect against heart disease. On the other hand, heavy drinking raises blood pressure and puts people at risk of stroke and heart failure.

Heavy alcohol use can result in appetite loss, sexual impotence or menstrual irregularities, vitamin deficiencies and infections. Alcohol irritates the lining of the stomach, which can be painful and is potentially fatal. Alcoholic liver disease is a major cause of illness and death in North America. Alcohol also increases the risk of liver, throat, breast and other cancers.

Chronic use of alcohol can damage the brain, which can lead to dementia, difficulties with co-ordination and motor control, and loss of feeling or painful burning in the feet. Alcohol dependence often results in clinical depression, and the rate of suicide among people who are alcohol-dependent is six times that of the general population.

Although women's average lifetime alcohol intake is less than half that of men, women are just as likely as men to develop alcohol-related diseases, and are twice as likely to die from these conditions.

Alcohol and the law

Provincial and federal laws regulate the manufacture, distribution, importation, advertising, possession and consumption of alcohol.

In Ontario it is illegal for anyone under 19 years of age to possess, consume or purchase alcohol; it is also illegal to sell or supply alcohol to anyone known to be or appearing to be (unless that person has proof otherwise) under the age of 19, or to sell or supply alcohol to anyone who appears to be intoxicated. Anyone who sells or supplies alcohol to others may be held civilly liable if people (including patrons of a tavern or restaurant and guests in a private home) injure themselves or others while intoxicated.

Federal criminal law sets out a range of drinking and driving offences. For more information, see "Do You Know. . . Alcohol, Other Drugs and Driving."

One in a series…

- Alcohol
- Alcohol, Other Drugs and Driving
- Amphetamines
- Anabolic Steroids
- Benzodiazepines
- Caffeine
- Cannabis
- Cocaine
- Ecstasy
- GHB
- Hallucinogens
- Heroin
- Inhalants
- Ketamine
- LSD
- Methadone
- Methamphetamine
- Opioids
- Rohypnol
- Tobacco

For more information on addiction and mental health issues, or a copy of this brochure, please contact the Centre for Addiction and Mental Health's 24-hour Information Line:
ONTARIO TOLL-FREE: 1 800 463-6273
TORONTO: 416 595-6111

To order multiple copies of this brochure, or other CAMH resource materials, please contact:
Marketing and Sales Services
TEL.: 1 800 661-1111 or 416 595-6059 in Toronto
E-MAIL: marketing@camh.net

To make a donation, please contact:
Centre for Addiction and Mental Health Foundation
TEL.: 416 979-6909
E-MAIL: foundation@camh.net

If you have questions, compliments or concerns about services at CAMH, please call our Client Relations Co-ordinator at:
TEL.: 416 535-8501 ext. 2028

Visit our Web site at: www.camh.net

Copyright © 2003 Centre for Addiction and Mental Health

Centre for Addiction and Mental Health
Centre de toxicomanie et de santé mentale

A Pan American Health Organization / World Health Organization Collaborating Centre
Affiliated with the University of Toronto

Disponible en français   2502 / 11-03   P251

Do You Know...

Alcohol

Street names: booze, sauce, drink

What is it?

Alcohol is a "depressant" drug. That means it slows down the parts of your brain that affect your thinking and behaviour, as well as your breathing and heart rate. The use of alcohol has been traced as far back as 8000 BC, and is common in many cultures today.

Where does alcohol come from?

Alcohol is produced by fermenting, and sometimes distilling, various fruits, vegetables or grains. Fermented beverages include beer and wine, which have a maximum alcohol content of about 15 per cent. Distilled beverages, often called "hard liquor" or "spirits," such as rum, whiskey and vodka, have a higher alcohol content.

Although alcohol comes in different forms, it has the same effect. In the following table, each "standard" drink contains 13.6 grams of alcohol.

Centre for Addiction and Mental Health
Centre de toxicomanie et de santé mentale

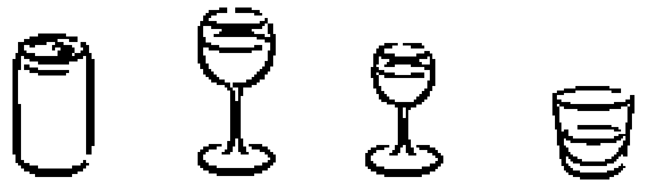

341 mL (12 oz.) beer* (5% alcohol)	=	142 mL (5 oz.) wine (12% alcohol)	=	85 mL (3 oz.) fortified wine** (16–18% alcohol)	=	43 mL (1.5 oz.) liquor (40% alcohol)

*Note that regular beers have an average alcohol content of five per cent, but some have as much as six or seven per cent, making them stronger than a "standard" drink. "Light" beers have an average alcohol content of four per cent.

**such as sherry, port or vermouth

What does it look like?

Pure (ethyl) alcohol is a clear, colourless liquid. Alcoholic beverages get their distinctive colours from their other ingredients, and from the process of fermentation.

Who uses alcohol?

Research reports that alcohol use among Ontarians age 18 and older was 87 per cent in 1992 and 79 per cent in 1999. Even though our laws restrict alcohol use to those 19 years of age and over, a 2001 survey of Ontario students in grades 7 to OAC found that 66 per cent reported using alcohol in the past year, and 27 per cent had been drunk at least once in the past four weeks.

In general, men drink more than women do, and are almost twice as likely to have drinking problems.

How does alcohol make you feel?

The way alcohol affects you depends on many factors, including:

- your age, sex and body weight
- how sensitive you are to alcohol
- the type and amount of food in your stomach
- how much and how often you drink
- how long you've been drinking
- the environment you're in
- how you expect the alcohol to make you feel
- whether you've taken any other drugs (illicit, prescription, over-the-counter or herbal).

For many people, a single drink of alcohol releases tension and reduces inhibition, making them feel more at ease and outgoing. Some people feel happy or excited when they drink, while others become depressed or hostile. Suicide and violent crimes often involve alcohol.

Women are generally more sensitive to the effects of alcohol than men, and all adults become increasingly sensitive to alcohol's effects as they age. When someone is more sensitive, it takes less alcohol to cause intoxication, and more time for the body to eliminate the alcohol consumed.

Early signs of alcohol intoxication include flushed skin, impaired judgment and reduced inhibition. Continued drinking increases these effects, and causes other effects, such as impaired attention, reduced muscle control, slowed reflexes, staggering gait, slurred speech and double or blurred vision. A severely intoxicated person may "black out," and have no memory of what was said or done while drinking. Effects of extreme intoxication include inability to stand, vomiting, stupor, coma and death.

How long does the feeling last?

It takes about one hour for the liver of a person weighing 70 kg (154 lbs.) to process and eliminate eight to 10 grams of alcohol, or about two-thirds of the alcohol contained in a standard drink. This rate is constant, no matter how much alcohol has been consumed, or what food or non-alcoholic beverages are taken.

Drinking heavily usually results in a "hangover," beginning eight to 12 hours after the last drink. Symptoms can include headache, nausea, diarrhea, shakiness and vomiting. A hangover is caused in part by acetaldehyde, a toxic chemical that is created as alcohol is processed by your liver. Other causes include dehydration and changes in hormone levels.

Some people think that having a drink before bed helps them to get to sleep. While alcohol does bring on sleep more quickly, it disturbs sleep patterns, and causes wakefulness in the night.

Is alcohol dangerous?

Yes, alcohol can be dangerous in a number of ways.

The impact of alcohol's effect on judgment, behaviour, attitude and reflexes can range from embarrassment, to unwanted or high-risk sexual contact, to violence, injury or death. Alcohol is involved in more regrettable moments, crimes and traffic fatalities than all other drugs of abuse combined. Young people, who are less familiar with the effects of alcohol, may be especially prone to act in an impulsive or dangerous manner while intoxicated.

Extreme intoxication can kill, often as the result of the person "passing out," vomiting and choking. A person who has been drinking heavily and is unconscious should be laid on his or her side and watched closely. Clammy skin, low body temperature, slow and laboured breathing and incontinence are signs of acute alcohol poisoning, which can be fatal. Seek emergency medical care.

Women who drink during pregnancy risk giving birth to a baby with behaviour problems, growth deficiency, developmental disability, head and facial deformities, joint and limb abnormalities and heart defects. The risk of bearing a child with these birth defects increases with the amount of alcohol consumed. The first trimester may be a time of greatest risk for the fetus, although there is no time during pregnancy when it is known to be safe to drink alcohol.

Mixing alcohol with other drugs — prescribed or recreational — can have unpredictable results. Alcohol may either block the absorption of the other drug, making it less effective, or it may increase the effect of the other drug, to the point of danger. The general rule is to never mix alcohol with any other drugs; for exceptions, ask your doctor.

Is there a safe level of drinking?

While there is no precise "safe" level of drinking, there are guidelines for adults who wish to lower the risks of drinking. People who are pregnant, who have certain medical conditions such as liver disease or mental illness, or who will be driving a vehicle or operating machinery, should avoid alcohol.

The "low-risk drinking" guidelines suggest spacing drinks an hour apart, and drinking no more than two standard drinks per drinking occasion. Men should have no more than 14 drinks a week, and women no more than nine.

Is alcohol addictive?

It can be.

Most alcohol-related illnesses, social problems, accidents and deaths are caused by "problem drinking." This term describes alcohol use that causes problems in a person's life, but does not include physical dependence. Problem drinking is four times as common as severe alcohol dependence.

Alcohol
